# Using Synthetic Datasets to Aggregate Data in Aging and Longevity Studies: A Randomized Resampling Approach

**DOI:** 10.1093/geroni/igaf122.4231

**Published:** 2025-12-31

**Authors:** Sarah Peskoe, Zachary Kunicki

**Affiliations:** Duke University, Durham, North Carolina, United States; Warren Alpert Medical of Brown University, Providence, Rhode Island, United States

## Abstract

The scarcity of harmonized datasets capturing clinical and cognitive outcomes—such as pain, function, biological, and behavioral measures—in older adults presents a major barrier to reproducible aging research. While federated data models offer one solution, they are often constrained by institutional privacy policies and logistical complexity. This study explores an alternative: pooled synthetic datasets generated using the synthpop package in R, which employs Classification and Regression Trees (CART) to model conditional distributions across mixed data types. We evaluate the utility of this approach using data from the Health and Retirement Study (HRS), focusing on how the choice of synthesis order affects bias and inference. Our findings show that fixed synthesis chains can introduce bias in downstream variables, while varying the chain improves accuracy for key outcomes. We propose a randomized resampling strategy to mitigate these effects, demonstrating improved precision and reduced bias in simulation. This work highlights the potential of synthetic data to support scalable, privacy-preserving, and methodologically rigorous research in aging and longevity, and proposes guidance and strategies to improve the statistical performance and integrity of synthetic datasets.

